# Genotype-Phenotype Characterization of Wolf-Hirschhorn Syndrome Confirmed by FISH: Case Reports

**DOI:** 10.1155/2012/878796

**Published:** 2012-11-22

**Authors:** F. Sheth, O. R. Akinde, C. Datar, O. V. Adeteye, J. Sheth

**Affiliations:** ^1^FRIGE's Institute of Human Genetics, FRIGE House, Satellite, Ahmedabad 380015, India; ^2^Sahyadri Medical Genetics and Tissue Engineering Facility, 1170/01, Barve Memorial Complex, J.M. Road, Pune 411005, India

## Abstract

The Wolf-Hirschhorn syndrome (WHS) is a multiple malformation and contiguous gene syndrome resulting from the deletion encompassing a 4p16.3 region. A microscopically visible terminal deletion on chromosome 4p (4p16→pter) was detected in Case 1 with full blown features of WHS. The second case which had an interstitial microdeletion encompassing *WHSC 1* and *WHSC 2* genes at 4p16.3 presented with less striking clinical features of WHS and had an apparently “normal” karyotype. The severity of the clinical presentation was as a result of haploinsufficiency and interaction with surrounding genes as well as mutations in modifier genes located outside the WHSCR regions. The study emphasized that an individual with a strong clinical suspicion of chromosomal abnormality and a normal conventional cytogenetic study should be further investigated using molecular cytogenetic techniques such as fluorescence *in situ* hybridization (FISH) or array-comparative genomic hybridization (a-CGH).

## 1. Introduction

The Wolf-Hirschhorn syndrome (WHS) is a well-known, multiple malformation syndrome, which affects 1 in 50,000 live births with a 2 : 1 female-to-male ratio [1, 2]. WHS is caused by a partial loss of genetic material from the distal portion of the p arm of chromosome 4 and is considered as a contiguous gene syndrome [3]. About 50–60% of the individuals with WHS have a microscopically visible *de novo* deletion encompassing a 4p16.3 region. The remaining 40%–45% have an unbalanced translocation, where as nearly as 55% can be detected by conventional banding techniques alone. These deletions may be *de novo* or inherited from a parent with a balanced rearrangement [4, 5]. In more than 95% of the cases, these deletions are diagnosed by fluorescent *in situ *hybridization (FISH) using Wolf-Hirschhorn syndrome critical region (WHSCR) specific probes.

WHS has attracted considerable attention and is associated with a variety of clinical features ranging from mild to severe mental retardation, hypotonia, growth delay, seizures, and specific craniofacial manifestations [2]. Some of these individuals do not display features consistent with WHS, whereas others have a clinical presentation with some overlap to the WHS phenotype. 

Deletion in the WHSCR regions have been considered as the hallmark of WHS. Mapping efforts have identified two different sized overlapping deletions defining the Wolf-Hirschhorn syndrome critical region 1 and 2 (WHSCR 1 and 2) [2, 6, 7]. These regions are suggested as being responsible for at least two of the core clinical manifestations of WHS—the developmental delay and the facial gestalt [8].

In this paper, we present 2 cases: deletion of a genomic segment on chromosome 4(p16→pter) in Case  1 with a loss of multiple overlapping genes and in another case a deletion encompassing WHSCR 1 and 2. These patients are clinically suspected with severe and mild WHS features, respectively.

**Case 1**

An eight-month-old girl presented with dysmorphic features, developmental delay, and mental retardation. She was the fifth child born to nonconsanguineous parents. The mother had an uneventful pregnancy. The father was 36 years old and the mother was 32 years old at the time of the delivery. The baby was born with low birth weight of 1.8 kg. At the age of 8 months she was admitted to the hospital due to the failure to thrive and was later found to have left side massive pneumothorax and cyanotic changes secondary to ASD with left-to-right shunt.

**Figure 1:**
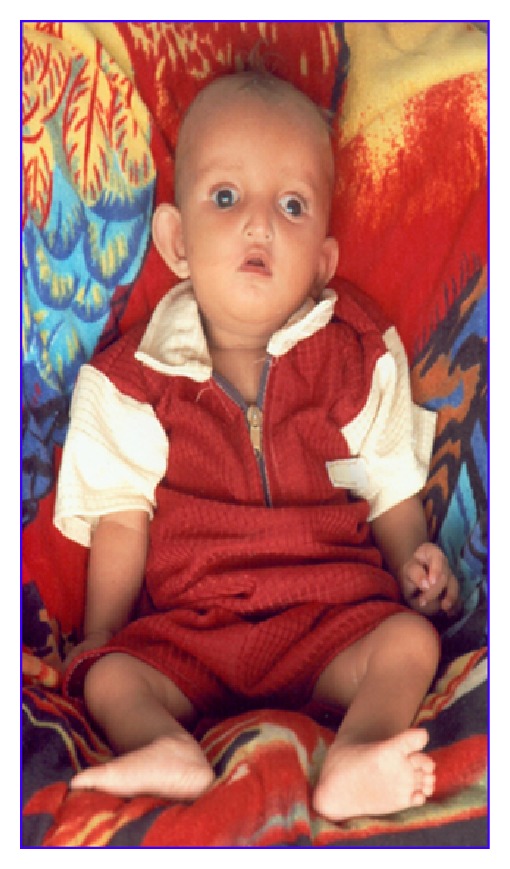
Depicting phenotypes of Case  1.

The clinical examination revealed marked growth retardation, microcephaly, prominent glabella, short philtrum, micrognathia, high forehead, preauricular tags with low set ears, narrow external auditory canals, strabismus, hypertelorism, iris coloboma, wide nasal bridge, downturned corners of the mouth with a fish-type appearance, and hyper convex fingernails (Figure 1).

**Case 2**

A four-year-old boy, the first child born to young and healthy nonconsanguineous parents with birth weight of 2.7 kg, was born at term by vaginal delivery. The age of the father was 33 years and the mother was 22 years at the time of the child's birth. The child presented with ophthalmic abnormalities, dysmorphic features and, delayed eye opening at neonatal period and was referred for clinical assessment.

**Figure 2:**
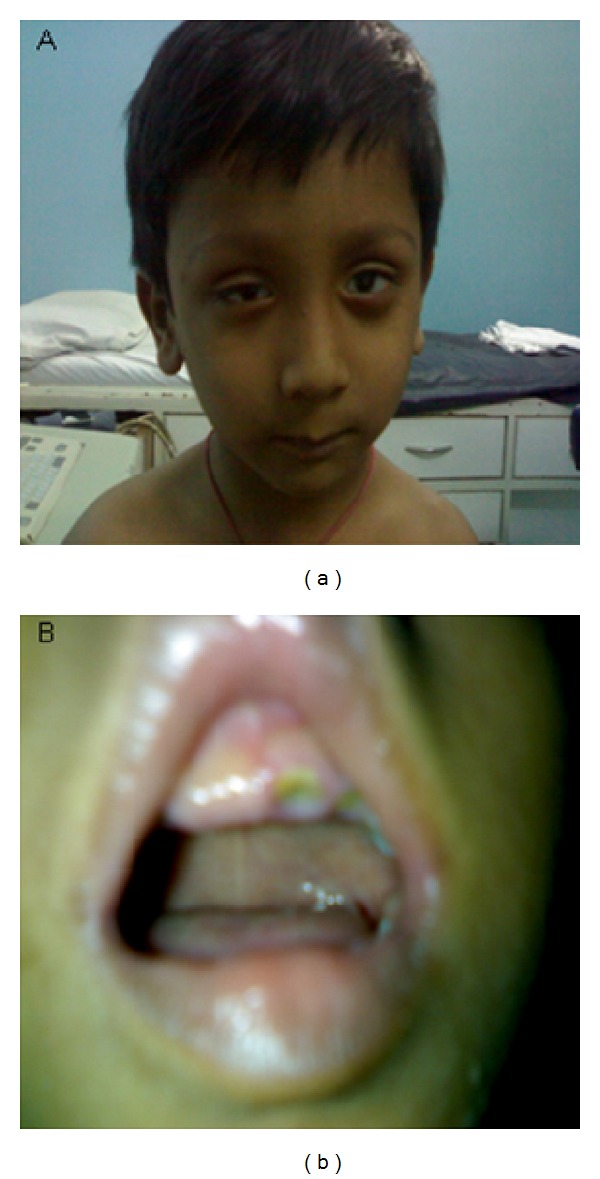
Facial feature of Case  2, (a) showing facial dysmorphism and (b) high arched palate and dental carries.

The clinical examination of this patient revealed relative macrocephaly, prominent glabellas, narrow palpebral fissures, microophthalmia, microcornea, coloboma of iris, hypertelorism, low set ears, broad nasal root with high arched palate, hypodontia and dental caries (Figure 2). Nystagmus was noted on the clinical examination and the visual acuity was reduced to 1 meter. The B-scan showed uveal coloboma, posterior vitreous detachment, retinal detachment, macular scar in the left eye, and ahpakia in the right eye in addition to the above findings. Supplementary investigations showed very mildly affected cognition, normal growth, and development of the CNS with no neurological deficits. Systemic examinations were observed to be normal.

**Figure 3:**
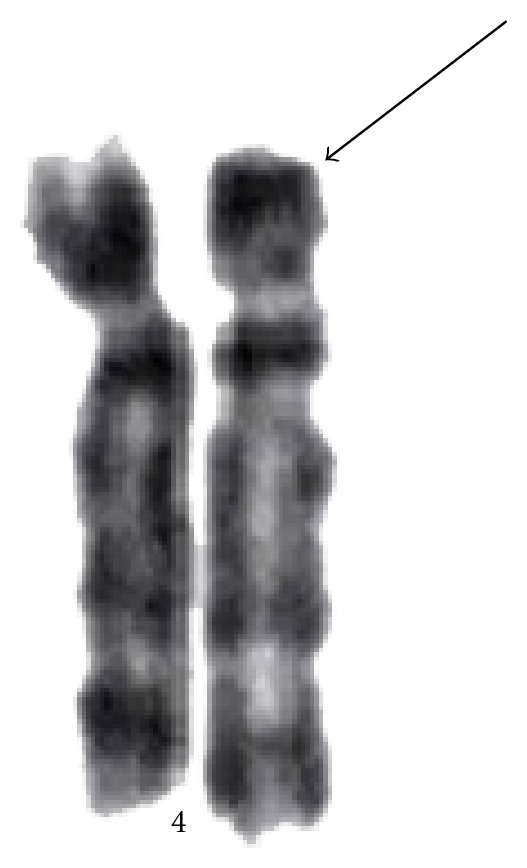
Arrow showing deletion at chromosome 4p16.

Cytogenetic study was carried out using peripheral blood lymphocytes by GTG banding according to the standard procedures at 550 band resolution and 25 metaphases were analyzed and karyotyped as per ISCN guidelines [9]. Metaphase analysis of Case  1 revealed a female karyotype, of 46,XX,der(4)del(4)(p16→pter) and that of Case  2 revealed a normal male karyotype, that is, 46,XY. Parents were chromosomally normal confirming *de novo* origin in both cases (Figure 3).

A submicroscopic deletion study was performed using the fluorescence *in situ* hybridization (FISH) analysis on the cytogenetic preparations using Kreatech dual colour probes. WHSC1 probe encompassing *WHSC1* gene was labeled with spectrum orange and control probe SE 4 was labeled with spectrum green as per manufacturer's recommendation. Locus specific BAC probe (RP11-262P20) covering *WHSC 1* and *WHSC 2 *genes was labeled with spectrum orange and a control BAC probe (RP11-195L6), positioned at 4q26, was labeled with spectrum green. Various overlapping BAC clones were used to narrow down the break site on chromosome 4 and were labeled as per the Vysis protocol as described by Menten et al. [10]. The signals were visualized by digital imaging microscopy and pseudocolouring was carried out using Adobe photoshop.

## 2. Discussion

Characteristic facial appearance and intellectual disability are the two major phenotypic features that constitute one of the major diagnostic markers for WHS. WHS is most often caused by terminal deletions involving chromosome 4p16.3 and may extend as far as 4p14 [11]. Interstitial deletions are less frequently reported [6, 7, 12], but are of a particular interest since they facilitate genotype–phenotype correlations and thus may aid in the search for causative genes.

The variability of WHS presentation has been attributed to the size of deletions. Zollino et al. [2] defines a patient with deletion between 5–18 Mb as “Classic WHS”, which presents with severe psychomotor delay and commonly has major malformations. Case  1 having 8.2 Mb deletion is consistent with several features reported in the literature as diagnostic markers for major WHS, which include microcephaly, mental retardation, growth retardation, high forehead, downturned mouth, hypotonia, congenital heart defects, coloboma, and dysplastic ears. Moreover, other features such as beaked nose, short philtrum, hypertelorism, nystagmus, coloboma, and prominent glabella are reported to be of low frequency as shown in Table 1. The variation in the size of the deleted segment and the effect of gene interaction might explain the absence of other reported phenotypes of WHS in this patient [13]. The phenotypic severity in this case is consistent with the length of deletion involving the WHSCR 1, WHSCR 2, and beyond (4p16→pter). The deleted segment includes all the genes involved in the development of the core features of WHS and other multiple genes that act as master regulators of different developmental pathways. Typically, the *MSX1* gene located at 4p16.2, outside the WHSCR 1 and 2, is deleted when the microscopically visible deletion involving 4p is involved. Haploinsufficiency of *MSX1* gene probably disrupts the regulation of several associated genes particularly involved in the development of the mouth, teeth, and the facial dysmorphisms [14, 15]. Hence, facial dysmorphism observed in Case  1 could be partly attributed to the loss of *MSX1* gene. This further supports the fact that the interaction with surrounding genes and mutations in modifier genes located outside the WHSCR regions account for the increase in phenotypic expression found in WHS with larger deletion. The plausible candidate gene for a part of craniofacial phenotype of WHS has been traced to *FGFR1 *[16]. Case  2 has a deletion of ~193 kb and presents with less striking features of WHS, such as mild retardation and fluent language without major malformation as compared to Case  1. This is consistent with Zollino's classification of “mild WHS” with a deletion of less than 3.5 Mb, with limited expression or absence of major malformations [2] as observed in Table 1. Milder phenotypic presentation has also been shown by South et al. in 2007 where the deletion region is small (1.78 Mb). The break point near or within the region of WHSCR gene and harbors *LETM1* may play a direct role in the seizure development if it gets deleted which has not been detected in our case [7]. Both WHSCR 1 and 2 were deleted in both cases. Case  1 has deletions that encompass beyond the critical regions to the telomeric end, while Case  2 has interstitial deletion involving only the WHSCR 1 and 2 and, hence, the variation in presentation. However, because of the deletion of WHSCR 1 and 2 in both cases, demonstration of some of the features of WHS, which includes high forehead, broad beaked nose, hypotonia, dysplastic ears, hypertelorism, coloboma, prominent glabella and nystagmus, are found in both cases, while dental caries, narrow palpebral fissure, microophthalmia and microcornea were only demonstrated in Case  2. The facial dysmorphism seen in Cases  1 and 2 could be due to the loss of several gene functions owing to both deletion and dysregulation. 

Hannes et al. [17] demonstrated 3D facial capturing on patients suggestive for WHS with deletions either located distally to the WHSCR 1 and/or overlapping the WHSCR 1 using dense surface modeling and pattern recognition technique. Full complement of the WHS facial characteristics was noted in typical WHS patients as seen in the two cases of this study. Interestingly, none of these patients were reported to have cleft palate as compared to Hannes et al. [17] findings. Hammond et al. [16] identified a 432 kb deletion located 600 kb proximal to both WHSCR 1 and 2 in a patient with a WHS phenotype and found that there are seven genes underlying this deleted region. It was hypothesized that the loci within WHSCR 1 and 2 exert long range effects on the deleted region. Thus in an event of WHSCR deletion, there will be dysregulation of the genes in a defined window or loci additive to the WHS critical regions leading to the increased phenotypic expression. The interplay of multiple genes in the deleted region and surroundings genes is essential for the expression of complete distinct facial phenotype seen in WHS patients. However, the reasons for the presence of certain phenotypes in Case  2 which are absent in Case  1 remain to be explained.

Conclusively, this study has shown that WHS encompasses a spectrum of phenotypes. Most likely to be missed is a microdeletion that presents milder or less striking clinical features and has apparently a “normal” karyotype. These will need molecular cytogenetic techniques (FISH/array comparative genomic hybridization), for further confirmation and following this, the inheritance pattern needs to be confirmed by studying parents and prenatal diagnosis can be offered accordingly.

## Figures and Tables

**Table 1:** Comparison of phenotypes in the cases and frequency of each phenotype in literatures.

Clinical Features	Wolf-Hirschhorn phenotypes 4p16.3	Case 1 4p16-pter (8.5 Mb)	Case 2 4p16.3 (3.5 Mb)	Frequency of phenotypes 4p15.32–4p16.3 [3, 12].
Dysplastic ears	+	+	+	80%
Hypotonia	+	+	+	75%
High forehead	+	+	+	50%
Colobomata of iris	+	+	+	40%
Broad or beak nose	+	+	+	?
Hypertelorism	+	+	+	?
Prominent glabella	+	+	+	?
Microcephaly	+	+	−	90%
Mental retardation	+	+	−	75%
Growth retardation	+	+	−	75%
Congenital defects (ASD)	+	+	−	50%
Micrognathia	+	+	−	?
Short philtrum	+	+	−	?
Preauricular tags	+	+	−	?
Downturned corners of the mouth with fish-type appearance	+	+	−	?
High arched palate	+	−	+	30%
Nystagmus	−	+	+	?
Hyper convex finger nails	−	+	−	60%
Dental caries	−	−	+	50%
Narrow palpebral fissures	−	−	+	50%
Microcornea	−	−	+	40%
Strabismus	−	+	−	?
Micro-ophthalmia	−	−	+	?
Seizuires	+	−	−	93%
Feeding difficulty	+	−	−	75%
Skeletal anomalies	+	−	−	60%
Renal anomalies	+	−	−	40%
Hypospadias	+	−	−	40%
Epicanthus	+	−	−	?
